# Increased risk for age-related impairment in visual attention associated with mild traumatic brain injury: Evidence from saccadic response times

**DOI:** 10.1371/journal.pone.0171752

**Published:** 2017-02-06

**Authors:** Jamie N. Hershaw, David M. Barry, Mark L. Ettenhofer

**Affiliations:** 1 Department of Medical and Clinical Psychology, Uniformed Services University of the Health Sciences, Bethesda, MD, United States of America; 2 Department of Behavioral Health, Madigan Army Medical Center, Tacoma, WA, United States of America; Kobenhavns Universitet, DENMARK

## Abstract

It was hypothesized that risk for age-related impairment in attention would be greater among those with remote history of mild TBI than individuals without history of head injury. Twenty-seven adults with remote history of mild TBI and a well-matched comparison group of 54 uninjured controls completed a computerized test of visual attention while saccadic and manual response times were recorded. Within the mild TBI group only, older age was associated with slower saccadic responses and poorer saccadic inhibition. Saccadic slowing was mitigated in situations where the timing and location of attention targets was fully predictable. Mild TBI was not associated with age-related increases in risk for neuropsychological impairment or neurobehavioral symptoms. These results provide preliminary evidence that risk for age-related impairment in visual attention may be higher among those with a history of mild TBI. Saccadic measures may provide enhanced sensitivity to this subtle form of cognitive impairment.

## Introduction

The normal aging process is characterized by a decline in cognitive function across many domains, including attention, beginning in early adulthood. Cognitive aging is purported to begin as early as the 20s, with a slow yet steady decline until older age (>60), at which time cognitive decline accelerates [1]. Individual differences in cognitive aging are partially attributable to the ability to adaptively use neural resources to perform cognitive functions as needed in response to brain damage or degeneration [2]. This is thought to be accomplished by reorganization of brain networks and functions, for example, by recruiting supplementary neural resources [3, 4]. In young and middle-aged adults, these mechanisms may preclude observable declines in cognition; however, cognitive decline may be exacerbated in individuals with a history of traumatic brain injury (TBI; [5, 6]). Reductions in brain volume, alterations in structural and functional connectivity, and neurotransmission dysfunction associated with moderate-to-severe TBI have been associated with poorer long-term cognitive outcomes [7–9]. Mild TBI has also been linked to greater age-related reductions in cortical thickness [10], and greater age-related alterations in neural activation [11]. Moreover, recent evidence suggests that mild TBI may influence the trajectory of cognitive aging. A study by Ozen and colleagues [12] found that older adults with a remote history of mild TBI performed worse on measures of executive function than older adults without a history of head injury. However, few studies have directly explored the potential impact of remote history of mild TBI on age-related changes in cognitive ability including young and middle-aged adults, or clinically-feasible methods measuring potential effects of mild TBI on the trajectory of cognitive aging.

Tests of visual attention may have particular value in the examination of combined effects of mild TBI and cognitive aging. In separate studies, mild TBI and increased age have each been associated with slower response time to visual stimuli [7, 13–16]. However, the combined effects of age and brain injury on different aspects of visual attention have remained largely unexamined. Research indicates that older adults have difficulty maintaining certain types of preparatory information (such as that provided by cues) to guide subsequent attention to a target [17, 18]. Performance of older adults on the Attention Network Test (ANT), an attention cuing paradigm [19], indicates that age differentially affects alerting processes (preparing for *when* a task-relevant stimulus will be present), spatial orienting processes (preparing for *where* a task-relevant stimulus will be present), and executive attention processes (resolving conflicting information about task-relevant stimuli). Older age has been associated with poorer alerting [20, 21] executive attention [22], and attentional disengagement, but not poorer spatial orienting [20–22]. Aging research consistently shows that bottom-up deficits are compensated by increased cortical activity, often in the prefrontal cortex [23–26]. This line of research suggests that, despite age-related decrements in attentional processing, normal behavioral performance is preserved through compensatory processing via changes in functional connectivity.

Similar to older adults, individuals with mild TBI frequently report attention problems and often perform poorly on tests of visual attention [27, 28]. Studies in acutely concussed patients have found impairments in spatial orienting and executive attention, while alerting remains unaffected [29, 30]. Moreover, evidence suggests that early deficits in spatial orienting recover within 7 to 14 days [31, 32] but executive deficits may persist for a longer period [29]. Adults with acute or chronic mild TBI also have greater difficulty with attentional disengagement (i.e., slower response time following release of attentional fixation prior to target onset, “gap effect”) than uninjured controls [33].

The overlap between effects of aging and mild TBI has a number of important implications. Older individuals who sustain mild TBIs during early adulthood may perform worse on tests of attention, memory, and executive function than their uninjured counterparts several decades after injury [34–36]. In one study, researchers demonstrated that middle-aged and older adults with remote history of mild TBI performed worse than uninjured older adults on tests of memory and attention, suggesting that that age-related cognitive decline occurred earlier in the clinical sample [37]. Additionally, electrophysiological indices of selective attention suggest impaired attentional processing among older adults with a history of mild TBI relative to uninjured controls [34]. Because mild TBI can disrupt functional connectivity [38, 39], it is possible that visual attention performance may be more vulnerable to the effects of age in individuals with a history of mild TBI relative to those without a history of head injury. Although previous studies provide some evidence that mild TBI may alter the trajectory of age-related changes in attention [12, 34, 37, 40], few have investigated this interaction in young and middle-aged adults and none to our knowledge has examined the combined effects of age and mild TBI across multiple attention processes or multiple response modalities. The purpose of this study is to examine the interactive effect of age and remote history of mild TBI on multiple attentional processes in a sample including young, middle-aged, and older adults.

In the present study, a novel cued attention paradigm, the Bethesda Eye and Attention Measure (BEAM; [41]) was used to assess visual attention in individuals with a history of mild TBI and uninjured controls. Using this task, multiple attention processes, including alerting, spatial orienting, interference control, attentional inhibition, attentional disengagement, and bottom-up attention were assessed using manual and saccadic response times to a variety of cue-target combinations. Previous research has demonstrated that this task elicits reliable and robust effects of alerting, spatial orienting, interference, inhibition, and gap signals within both saccadic and manual RT modalities [41]. Additionally, BEAM saccadic metrics appear to be particularly sensitive to history of remote mild TBI [42]. Given potential overlap between effects of mild TBI and age-related cognitive impairment, it was hypothesized that age-related differences in overall visual attention would be greater in individuals with a history of mild TBI relative to uninjured controls. Moreover, in light of research suggesting that both mild TBI and advanced age may impact interference control and inhibition (collectively termed “executive attention”) and attentional disengagement, it was expected that performance on trials eliciting attentional disengagement and executive attention will be more strongly related to age in individuals with remote history of mild TBI compared to uninjured controls.

## Method

### Participants

All study procedures were approved by the Uniformed Services University IRB; written informed consent was obtained from all participants. Participants and data from this study were derived from a parent study [42] conducted within a university research setting. The parent study examined overall saccadic impairment in participants with remote history of mild TBI as compared to uninjured controls; age was not examined as a primary variable. The current manuscript describes a follow-up study to examine the combined effects of age and mild TBI on task performance. One hundred ten participants greater than 18 years of age were recruited through advertisements in military, medical, research, and public settings in the greater Washington DC metro area. Individuals were included in study if they had a history of mild TBI (mild TBI group), defined as one or more injuries that resulted in a loss of consciousness (LOC) of less than 30 minutes and/or post traumatic amnesia (PTA) for less than 24 hours, or if they had no history of head injury (control group). Criteria for mild head injury are consistent with the criteria defined by the American Congress of Rehabilitation Medicine [43]. Information about head injury was obtained through a detailed structured interview and verified through consensus by a team including two licensed providers with post-doctoral training in assessment of head injury. The interview included questions pertaining to medical history, circumstances surrounding and mechanisms of the head injury, length of LOC, PTA, and other alterations of consciousness (AOC), such as confusion or disorientation. Twelve participants were excluded for history of moderate-to-severe TBI; 4 participants with history of head injury with AOC only; 7 participants for other medical conditions affecting cognitive performance; 2 individuals for failing two or more embedded neuropsychological measures of response validity; 3 participants for failing to follow task instructions, and one participant for whom technical difficulties interfered with eye tracking data acquisition.

After exclusion, 27 participants with a history of mild TBI and 54 uninjured controls were included in this study. Participant characteristics are presented in Table 1. In this sample of 81, 36 (44%) were male. The mean age was 33.75 years (SD = 11.77) and the mean years of education was 16.15 (SD = 2.47). The median number of years since injury in the mild TBI group was 6.91 years (*IQR* = 2.75, 21.86). Twenty participants in the mild TBI group (74%) sustained one head injury, whereas seven (26%) sustained two or more. The mild TBI group did not significantly differ from the control group on age, education, ethnicity, gender, or estimated premorbid intelligence.

**Table 1:** Participant Characteristics.

	Control	Mild TBI	*p*^a^
N	54	27	
Female	53.70%	59.26%	.64
Mean age in years (*SD*)	33.17 (11.42)	34.93 (12.59)	.53
Mean years education (*SD*)	16.20 (2.63)	16.04 (2.16)	.78
Mean estimated IQ (*SD*)	108.59 (11.23)	110.15 (9.21)	.54
Race / Ethnicity			.83
White	30	18	
Hispanic	3	2	
Asian	4	1	
Black	15	5	
Other	2	1	

a Statistical significance of t-test or chi-square, as appropriate.

### Procedure

After providing written informed consent, participants completed a structured interview to obtain demographic information, medical history, and a detailed history of head injury. Participants then completed a neuropsychological battery, followed by evaluation of visual attention with BEAM.

### Measures

#### Bethesda Eye and Attention Measure (BEAM) v.34

BEAM is a cued visual attention paradigm designed to concurrently measure saccadic and manual response time. BEAM is described in greater detail elsewhere [41]. Prior studies demonstrate that BEAM is able to reliably elicit and measure multiple elements of attention via saccadic and manual responses [41] and that these metrics are associated with global cognitive performance [42]. The following description will briefly summarize the task. Participants are instructed to make a saccade to a target and also press a button as quickly as possible upon target detection. There are six distinct cue-target combinations presented to participants constituting six unique trial types. The task consists of four blocks of 48 trials presented in pseudorandom order, for a total of 192 trials. Trial type is counterbalanced within each block with respect to cue type (described below) and target location. Total task duration is approximately 12 minutes plus calibration (approximately 15 minutes total).

At the start of a trial, a central fixation cross is presented psuedorandomly between 1500 and 2500ms and is then replaced by a central cue for 200ms, followed by a target. Cues are randomly selected to be a white arrow pointing left or right, a white diamond, or a red arrow pointing left or right. Valid white arrow cues point in the direction of the impending target for “directional” trials, while invalid white arrow cues point in the opposite direction of the impending target for “misdirectional” trials. “Non-directional” trials are cued with a white diamond. “No-go” trials consist of red arrows that are predictive of target location (i.e., 100% validity). Examinees are instructed to withhold a response when they see a red arrow. Two-hundred milliseconds following cue onset for all trials, a target appears either left, right, above, or below the cue. The cue remains on screen during the duration of the target. For “uncued” trials, no cue is presented and the fixation cross remains on screen. For “gap” trials, the central fixation cross disappears for 200ms and is not replaced by a cue. Targets, oriented either horizontally or vertically from center, remain onscreen for 1000ms, during which time participants are required to make a saccade to the target and press a button on a response box to indicate target detection, with the exception of no-go trials (red arrows), for which participants do not make a saccade or a button press. The trial ends when the target and cue go off-screen and are replaced by a fixation cross. Trial type is counterbalanced within blocks such that each trial type is presented a total of 32 times during the task.

Directional trials are designed to induce both spatial and temporal attention orienting (“orienting”), as they provide information regarding the location and timing of the upcoming target. Misdirectional trials are designed to induce cognitive control because the arrows incorrectly orient participants to the opposite location of the impending target, after which participants must inhibit their prepotent response and reorient to the correct location (“interference”). Non-directional trials are designed to elicit temporal attention orienting (“alerting”), as they alert participants to an upcoming target but do not contain spatial information. Gap trials are designed to release attentional engagement prior to the onset of a target by removing visual stimuli (“attentional disengagement”). Uncued trials provide neither spatial nor temporal advance information. They provide a measure of bottom-up, stimulus-driven visual attention. No-go trials were included to provide a metric for saccadic and manual attentional inhibition. Metrics derived from the uncued, gap, directional, misdirectional, and non-directional trials included saccadic response time (i.e., latency to fixate on target following target onset), manual response time (i.e., latency to press the button following target onset), and accuracy (i.e., fixation on the target and manual button press). The no-go trials yielded only accuracy data (i.e., inhibition of saccade towards target and inhibition of manual button press) from which a ratio of inhibition errors was computed.

#### Neuropsychological evaluation

A standardized neuropsychological battery was administered in order to facilitate comparison of BEAM results to more conventional measures of cognitive performance. Neuropsychological tests included: Grooved Pegboard (GP Dominant and Non-dominant Hand Score; [44]); Trail Making Test (TMT Parts A and B; [45]); Conners’ Continuous Performance Test–II (CPT-II Hit RT, Hit RT SE, Omissions, and Commissions; [46]); California Verbal Learning Test-II (CVLT-II Trial 1–5 Total, Short Delay Free Recall, Long Delay Free Recall; [47]); Wechsler Adult Intelligence Scale IV (WAIS-IV Digit Span Forward, Digit Span Backwards, Symbol Search Total Score; [48, 49]); Delis-Kaplan Executive Function System (D-KEFS Color Word Interference Test Trials 1, 2, 3, and 4 time; [50]). Results from these cognitive tests were standardized using published normative data and aggregated into scores representing global cognitive ability as well as individual cognitive domains of motor performance, processing speed, attention, learning/memory, and executive functions. While these tests are normed by age, it should be noted that this standardization process would not prevent the detection of potential differences in cognitive aging between groups. Additionally, premorbid verbal intelligence was estimated using the Wechsler Test of Adult Reading (WTAR; [51]). Embedded metrics from TMT, CPT-II, WAIS-IV Digit Span, and CVLT-II were used to evaluate performance validity (i.e., test-taking effort). The neurobehavioral symptom inventory (NSI; [52]) was used to measure self-reported neurobehavioral symptoms.

### Statistical analysis

Multivariate analyses of covariance (MANCOVAs) were performed to examine the interaction of age and mild TBI on BEAM attention metrics. Separate analyses were computed for each of the two response modalities: manual and saccadic. Dependent variables included response time for directional, misdirectional, non-directional, uncued, and gap trials, as well as the ratio of inhibition errors committed on the no-go trials. Group (mild TBI or control) was entered as a fixed factor and age was entered as a covariate. For any analysis demonstrating a significant age by group interaction, follow-up linear regressions were conducted separately for each group and trial type, with performance on each trial type as the dependent variable and age as the independent variable. Hierarchical linear regression was used to perform additional analyses examining potential interactions of age and mild TBI on global cognitive ability (as represented by the neuropsychological battery) and neurobehavioral symptoms. It should be noted that main effects of group (mild TBI vs. control), while not the primary focus of this study, are reported to support interpretation of interaction effects (mild TBI x age).

## Results

**Fig 1:**
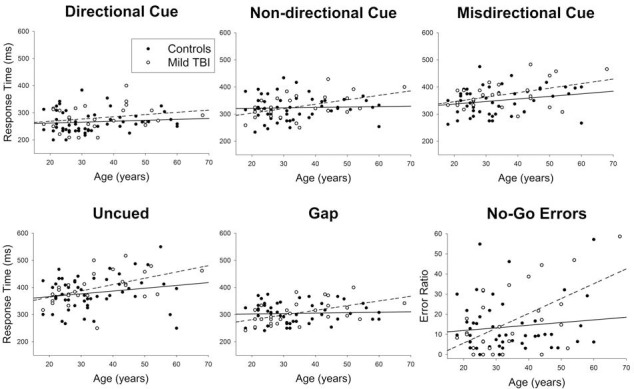
Linear regressions of age on individual BEAM trial types for controls (solid line) and mild TBI (dotted line).

A MANCOVA testing the interaction of age and mild TBI on saccadic task performance showed a significant main effect of group, F(6,72) = 2.386, *p* < 0.05, partial η^2^ = 0.166, Wilk’s λ = 0.834. Overall, the mild TBI group had longer saccadic response times and committed more no-go inhibition errors. Univariate tests showed that there were marginally significant group differences on ratio of no-go inhibition errors, F(1,77) = 3.686, *p* = .059, partial η^2^ = 0.046, and saccadic response time for gap trials, F(1,77) = 3.861, *p* = .053, partial η^2^ = 0.048. There was also a significant interaction between age and group, F(6,72) = 2.631, *p* < 0.05, partial η^2^ = 0.180, Wilk’s λ = 0.820. Results of the planned linear regressions are shown in are depicted in Fig 1. Age was not significantly associated with performance on any BEAM trial types among uninjured controls. However, within the mild TBI group, more advanced age was associated with slower saccadic RT on non-directional (β = 0.472, R^2^ = 0.223, *p* < 0.05), misdirectional (β = 0.423, R^2^ = 0.179, *p* < 0.05), gap (β = 0.514, R^2^ = 0.264, *p* < 0.01), and uncued (β = 0.497, R^2^ = 0.247, *p* < 0.01) trials. More advanced age was also associated with a greater proportion of saccadic inhibition errors committed during no-go trials (β = 0.567, R^2^ = 0.321, *p* < 0.01). Age was not significantly associated with saccadic RT on directional trials [β = 0.233, R^2^ = 0.054, *p* > 0.05].

A MANCOVA testing the interaction of age and mild TBI on manual task performance showed a significant effect of age, F(6,72) = 3.036, *p* < 0.05, partial η^2^ = 0.202, Wilk’s λ = 0.798. However, the effect of group, F(6,72) = 1.245, *p* > 0.05, and the interaction between age and group, F(6,72) = 0.702, *p* > 0.05, were not significant. Therefore, follow up regressions were not conducted for BEAM manual performance data.

In a hierarchical linear regression testing the interaction of age and mild TBI on global neuropsychological status, the addition of the interaction term did not account for significantly more variance than age and mild TBI alone, F(3,77) = 0.852, *p* > 0.05, R^2^-change(1,77) = 0.001, *p* > 0.05. Similarly, in a hierarchical linear regression testing this interaction on self-report symptoms, the addition of the interaction term to the model with age and mild TBI did not account for a significant amount of additional variance in total NSI score, R^2^-change(1,77) = 0.031, *p* > 0.05.

### Follow-up analyses

To evaluate whether the observed changes in RT may be influenced by a speed-accuracy trade-off, we submitted accuracy data to a 2 (group) X 5 (trial type: directional, non-directional, misdirectional, uncued, and gap) repeated measures ANOVA to evaluate any group differences in accuracy. There was an effect of trial type, in which uncued (M = 91.89, SD = 1.38) had significantly lower accuracy than directional (M = 93.92, SD = 1.17), and gap trials (M = 91.55) had significantly lower accuracy than directional and non-directional trials (M = 93.26, SD = 1.09). No group difference or interaction between group and trial type was observed.

## Discussion

Previous research suggests that a history of brain insult may exacerbate cognitive aging across a variety of domains [10, 12, 37]. The results of the current study provide additional evidence in support of this premise. Importantly, while much research has focused on memory, the current study highlights the combined influence of mild TBI and age on visual attention. Additionally, most research on cognitive aging investigates age effects in older adults; our study investigated age effects over the adult lifespan. As expected, age was more strongly related to saccadic measures of visual attention in adults with a history of remote mild TBI than in a well-matched sample of uninjured controls. Specifically, combined effects of age and mild TBI were noted in the form of slower saccadic response times and greater difficulty inhibiting saccadic responses. These results suggest that visual attention may be particularly vulnerable to the effects of age in adults with a history of mild traumatic brain injury. Consistent with our hypotheses and previous research [12, 53], saccadic metrics representing executive attention (i.e., those requiring response inhibition or resolution of misleading spatial information) showed greater age-related differences in the mild TBI group than in the uninjured control group. Combined effects of age and mild TBI were also present for saccadic metrics representing alerting (i.e., trials when a timing cue was provided), and for uncued trials representing bottom-up visual attention. In contrast, combined effects from mild TBI and age were not demonstrated for orienting trials, in which the timing and location of the target were both predictable. These results suggest that multiple aspects of visual attention may be affected by age among those with a history of mild TBI, but that these effects are not universal to all types of visual attention.

In comparison to findings from our saccadic measures, no age-related performance differences were demonstrated between the mild TBI and control groups on measures of global cognitive ability, self-reported neurobehavioral symptoms, or manual measures of visual attention. Potentially, brain networks supporting saccadic performance are at increased risk for disruption relative to brain networks supporting somatomotor performance. Alternately, it is possible that compensatory processes are less effective at mitigating impairments in the very rapid forms of processing required for intact saccadic performance than for parallel somatomotor responses, which generally occur at a slower time scale. Additionally, the difference in saccadic response times does not seem to reflect a change in cognitive strategy, as no differences in accuracy were observed. Prior literature on compensatory mechanisms of aging provides some indirect support for our results. Older adults typically have declines in attention processing; however, the effects of aging can be partially mitigated by compensatory activation across the cortex, particularly in the prefrontal cortex [24, 26, 54]. Healthy older adults may perform similarly on tasks but show different patterns of neural activation, indicating compensatory mechanisms sufficient to preserve normal behavior despite age-related changes in the brain. Our finding that the mild TBI group had stronger effects of age on saccadic performance suggests that the compensatory mechanisms commonly reported in healthy aging samples are not functioning optimally in mild TBI. This premise is supported by prior evidence indicating that more distributed functional connectivity and intact white matter tracts are positively associated with cognitive and behavioral outcomes [55, 56]. Given that the primary form of neuropathology attributed to mild TBI is axonal injury, resulting in reduced functional connectivity [38, 57, 58], mild TBI may render age-related compensatory mechanisms insufficient to preserve normal saccadic behavior, while other networks may benefit more from such compensatory mechanisms.

Combined effects of mild TBI and age were demonstrated for each of the test trials requiring inhibition, resolution of conflicting information, or response to unpredictable target locations; only performance on fully-predictable “directional” trials, arguably the easiest trial type, was unrelated to age in the mild TBI group. These findings suggest that, while older individuals with mild TBI may have difficulty compensating for deficits in saccadic performance, these vulnerabilities may be reduced under highly structured conditions with lower levels of cognitive load. These results are consistent with a theory postulated by Ghajar and Ivry [59] stating that mild TBI causes deficits of anticipatory activation. However, instead of being unable to adequately use advance information to predict the timing and location of a stimulus, as previously suggested, our results suggest that as adults with a history of mild TBI age, they may be more reliant on predictability in spatial location of visual information, and thus, when that information is absent or invalid, it causes processing deficits that are difficult to overcome (i.e., difficult for which to compensate).

Age was not associated with cognitive performance among uninjured controls. For the neuropsychological battery, which was standardized by age, this finding is consistent with expectation. However, the response time data do not reflect previous findings in the literature that indicate negative effects of age on attention [20, 21]. These results are likely related to sample characteristics, as our sample did not include enough older adults to detect these effects (age M = 33.75; range: 18–68; SD = 11.77). By comparison, previous studies demonstrating cognitive decline in healthy adults had samples averaging 70 to 80 years [20, 22]. Previous research has shown that age effects on RT tend to be modest in middle-aged individuals, accelerating more rapidly in old age [60]. Additional research using a larger sample with greater representation of older adults is needed to determine the trajectory of visual attention deficits across the lifespan, as our current study cannot address the effects of normal aging on saccadic response time.

Injury characteristics such as age at time of injury, time since injury, number of injuries, and injury severity may influence the outcome of the interaction of age and mild TBI on visual attention processing. However, sufficient sample size was not available in the current study to test the potential influence of these factors. Additional longitudinal research will be able to test the effects of aging within individuals and provide greater power to detect age-related changes within the same individuals over time. Thus, it is hoped that the current study represents an initial step in a larger effort to understand the nature of long-term effects of mild TBI on attention as individuals age. Our results indicating measurable age-related deficits in saccadic response time for mild TBI, but not in manual response time, self-report symptomatology, or global neuropsychological status, suggests that any differences in age-related cognitive impairment among those with a history of mild TBI are not universal to all aspects of cognitive performance or approaches to measurement. These results also have important implications for clinical assessment. Prior research in our lab has shown that saccadic responses are less affected by confounding characteristics such as intelligence than manual responses [41], and more sensitive to residual effects of remote mild TBI [42]. In the current analysis, we have further demonstrated that saccadic RT is better able to detect combined effects of brain injury and age. These findings suggest that traditional neurocognitive tasks (i.e., those measuring only somatomotor responses) and symptom self-reports may not be sensitive to subtle changes related to age in patients with remote mild TBI. Evaluation of patients with remote mild TBI might be improved if saccadic responses are included to derive additional cognitive performance metrics.

Importantly, our study is among the first to test combined effects of age and mild TBI on visual attention. This preliminary research suggests that age-related impairment in visual attention may be exacerbated in individuals with a history of mild TBI, even in young and middle-age. These cross-sectional findings are consistent with theories that head injury contributes to greater age-related cognitive decline, although these theories remain largely untested [6, 10, 12, 37]. While the conclusions from this study must be tempered by relatively small size and cross-sectional design, this study represents an important early step towards understanding the role of age in the long-term effects of mild TBI. Additional research that directly measures neural differences in attentional cue processing and compensatory mechanisms related to mild TBI is needed to further investigate these effects.
